# Donor age effects on *in vitro* chondrogenic and osteogenic differentiation performance of equine bone marrow- and adipose tissue-derived mesenchymal stromal cells

**DOI:** 10.1186/s12917-022-03475-2

**Published:** 2022-11-03

**Authors:** Jasmin Bagge, Lise Charlotte Berg, Jennifer Janes, James N. MacLeod

**Affiliations:** 1 Department of Veterinary Clinical Sciences, University of Copenhagen, Agrovej 8, 2630 Taastrup, Denmark; 2 Department of Veterinary Science, Maxwell H. Gluck Equine Research Center, University of Kentucky, 1400 Nicholasville Rd, Lexington, KY 40546 USA; 3 Department of Veterinary Science, University of Kentucky Veterinary Diagnostic Laboratory, University of Kentucky, 1490 Bull Lea Rd, Lexington, KY 40511 USA

**Keywords:** Horse, Donor age, Aging, Mesenchymal stromal cells, Cartilage, Bone

## Abstract

**Background:**

Bone marrow (BM)- and adipose tissue (AT)-derived mesenchymal stromal cells (MSCs) have shown potential as cell-based therapies for cartilage and bone injuries and are used increasingly in human and veterinary practice to facilitate the treatment of orthopedic conditions. However, human and rodent studies have documented a sharp decline in chondrogenic and osteogenic differentiation potential with increasing donor age, which may be problematic for the important demographic of older orthopedic patients. The aim of this study was to identify the effect of donor age on the chondrogenic and osteogenic differentiation performance of equine BM- and AT-MSCs in vitro.

BM- and AT-MSCs and dermal fibroblasts (biological negative control) were harvested from horses in five different age groups (*n* = 4, *N* = 60); newborn (0 days), yearling (15–17 months), adult (5–8 years), middle-aged (12–18 years), and geriatric (≥ 22 years). Chondrogenic differentiation performance was assessed quantitatively by measuring pellet size, matrix proteoglycan levels, and gene expression of articular cartilage biomarkers. Osteogenic differentiation performance was assessed quantitatively by measuring alkaline phosphatase activity, calcium deposition, and gene expression of bone biomarkers.

**Results:**

Chondrogenic and osteogenic differentiation performance of equine BM- and AT-MSCs declined with increasing donor age. BM-MSCs had a higher chondrogenic differentiation performance. AT-MSCs showed minimal chondrogenic differentiation performance in all age groups. For osteogenesis, alkaline phosphatase activity was also higher in BM-MSCs, but BM-MSCs calcium deposition was affected by donor age earlier than AT-MSCs. Chondrogenic and osteogenic differentiation performance of BM-MSCs exhibited a decline as early as between the newborn and yearling samples. Steady state levels of mRNA encoding growth factors, chondrogenic, and osteogenic biomarkers were lower with increasing donor age in both MSC types.

**Conclusions:**

The data showed that chondrogenic and osteogenic differentiation performance of equine BM-MSCs declined already in yearlings, and that AT-MSCs showed minimal chondrogenic potential, but were affected later by donor age with regards to osteogenesis (calcium deposition). The results highlight the importance of donor age considerations and MSC selection for cell-based treatment of orthopedic injuries and will help inform clinicians on when to implement or potentially cryopreserve cells. Moreover, the study provides molecular targets affected by donor age.

**Supplementary Information:**

The online version contains supplementary material available at 10.1186/s12917-022-03475-2.

## Background

Orthopedic injuries are a major cause of loss-of-function, pain, and morbidity in humans and horses worldwide leading to substantial personal and financial losses [1–3]. Articular cartilage injuries are particularly problematic, since cartilage has a very limited intrinsic repair capacity, believed to be due in part to a lack of vasculature and low mitotic activity of the chondrocytes [4]. Lesions involving articular cartilage often include pathology of the subchondral bone, causing significant pain and long periods of rest with a poor prognosis to return to full function [4, 5]. Unlike articular cartilage, bone is vascularized and has access to progenitor cells which in general provides for better healing capabilities. Nevertheless, a persistent reparative response with higher bone resorption than bone formation has been reported in horses. This leads to an architectural disruption and weak areas associated with subchondral bone lesions [6]. Persistent bone resorption areas are therefore believed to be one of the contributing factors to osteoarthritis [6]. Consequently, there is great need for articular cartilage and bone restoration methods.

Unfortunately, none of the existing techniques have been able to fully restore articular cartilage structure and function with complete integration into the normal surrounding tissue [5, 7–9]. Cell-based therapies with multipotent mesenchymal stromal cells (MSCs) are an area of high scientific and clinical interest. MSCs have shown capacity to facilitate repair of orthopedic injuries through immunomodulation, growth factor secretion, and cellular differentiation in addition to alleviating symptoms [8, 10, 11]. Clinically relevant MSC tissue sources include bone marrow (BM) and adipose tissue (AT), where published literature has reported promising osteogenic and chondrogenic capacity [12–15]. Efforts to direct and improve MSC differentiation potential includes different cellular matrices and incorporation techniques, where scaffolds enhance retention of the MSCs within the injury site, support cell growth and differentiation, and improve tissue regeneration [16, 17]. Currently, cell choice is frequently influenced by existing precedence, convenience of sampling, cost, and commercial interests, in addition to the consideration of which MSC type has the better biological potential for regenerating the injured tissue. Several studies have compared chondrogenic and osteogenic differentiation in a range of MSCs, and most studies report a higher potential of BM-MSCs compared to AT-MSCs [14, 18–20]. However, human and rodent studies have shown a sharp decline in MSC quantity and quality with increasing donor age [21–23]. Orthopedic injuries are prevalent in older patients [3, 24], making donor age an important variable in cell therapy. Most MSC donor age studies have been performed only on BM-MSCs [21–23, 25, 26]. Asumda et al*.* showed that BM-MSCs from 15 month old rats had lost their chondrogenic and osteogenic differentiation potential [21], which was supported by a 76% decline in osteogenic alkaline phosphatase activity in adult human BM-MSCs [26], and an 82% decline in proteoglycan content of chondrogenic induced BM-MSC pellets from 1 year old rats compared to 1 week old rats [25]. A comparative study in mice reported that osteogenesis was less affected by donor age in AT-MSCs than BM-MSCs [27], but there is a clear lack of knowledge on whether these different sources are affected equally by donor age. No donor age studies have been reported in equine BM- and AT-MSCs with regards to chondrogenic and osteogenic differentiation performance.

The aim of this study was, therefore, to identify the effect of donor age on the chondrogenic and osteogenic in vitro differentiation performance of equine BM- and AT-MSCs. Our hypothesis was that increasing donor age is a major variable impacting chondrogenic and osteogenic in vitro differentiation performance in equine BM- and AT-MSCs with decreasing capacities following non-linear kinetics. Specific aims were 1) to compare pellet size, histological cell morphology and proteoglycan staining in chondrogenic induced cell pellets, and to compare calcium deposits and alkaline phosphatase activity in osteogenic induced monolayer cultures, and 2) to compare steady state mRNA expression of targeted genes specific to articular cartilage in cell pellets and bone in monolayer cultures.

## Results

BM-MSCs, AT-MSCs, and dermal fibroblasts (DF) (biological negative control) were analyzed from horses of mixed breeds across five different age groups. Age groups were newborn (0 days old), yearling (15–17 months), adult (5–8 year old), middle-aged (12–18 year old), and geriatric (≥ 22 year old). Four horses were used for each cell type and age group (*n* = 4, *N* = 60).

### Chondrogenesis

#### Pellet size

**Fig. 1:**
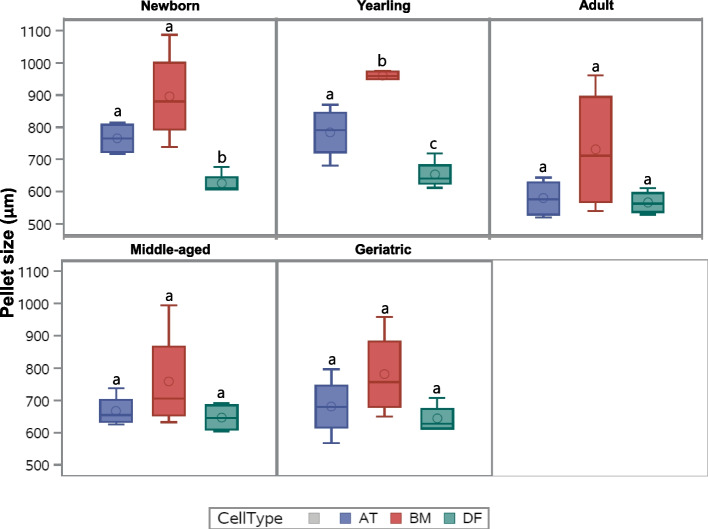
Pellet size of chondrogenic induced cells. Box plot showing pellet size (µm) of chondrogenic induced bone marrow (BM)- and adipose tissue (AT)-derived mesenchymal stromal cells and dermal fibroblasts (DF) in five different age groups (*n* = 4, *N* = 60). All pellets were generated from 5 × 10^5^ cells and cultured in chondrogenic induction medium for 21 days. Cell types within the same age group not labeled with the same letter are significantly different from each other (*p* < 0.05)

Chondrogenic-induced pellet size varied from ~ 0.5 mm to over 1 mm in mean diameter as shown in Fig. 1, with the following general size relationship: BM-MSC > AT-MSC > DF (*p* < 0.006). No effect of donor age was seen in BM-MSC pellets (Fig. 1). For AT-MSCs, pellets from adult horses were significantly smaller compared to newborn (*p* = 0.006) and yearlings (*p* = 0.01) (Fig. 1). Pellets from BM-MSCs were only significantly larger than AT-MSCs in yearlings (*p* = 0.004), whereas BM-MSC pellets were significantly larger than DF pellets in newborn (*p* = 0.02) and yearlings *(p* < 0.0001) (Fig. 1). Non-induced control pellets from yearling BM-MSCs had a diameter between 0.38 and 0.45 mm.

#### Morphological description of chondrogenic induced pellets

Hematoxylin and Eosin (H&E) stained pellets from each age group with the cell types (BM-MSCs, AT-MSCs, and DFs) were assessed microscopically with representative descriptions below.

BM-MSCs: Newborn, yearling, adult, and middle-aged pellets are composed of a central accumulation of polygonal cells with minimal acidophilic cytoplasm and round to oval nuclei often within lacunae separated by moderate amounts of lightly basophilic matrix. Few necrotic cells are interspersed within this population. This population of polygonal cells within lacunae is largest in the newborn pellets and decrease with age. The remaining portion of the pellets is composed of an outer rim of spindle cells that increase in thickness with age. The geriatric pellets are composed largely of small, polygonal cells with pyknotic nuclei bordered by a rim of spindle cells.

AT-MSCs: All age groups exhibited similar features. Pellets were composed of a varying sized central necrotic core. The remaining portion of the pellet is composed of fairly well organized dense streams of spindle cells.

DFs: All age groups exhibited similar features. Pellets were composed of varying sized central cores of necrotic cells bordered by fairly organized dense streams of spindle cells.

#### Proteoglycan staining

**Fig. 2:**
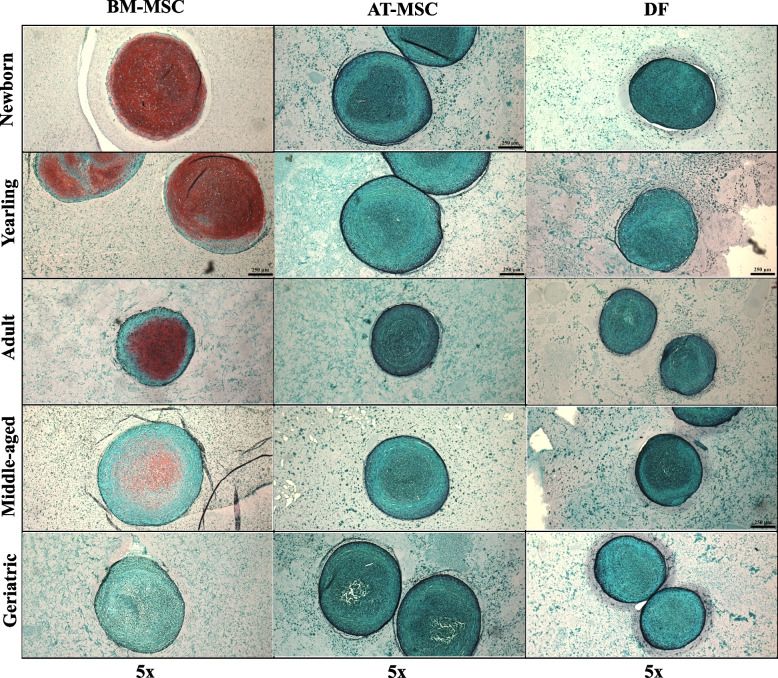
Safranin-O images of chondrogenic induced cells. Panel showing representative Safranin-O stained pellets of bone marrow (BM)- and adipose tissue (AT)-derived mesenchymal stromal cells (MSCs), and dermal fibroblasts (DF) from horses in five different age groups after 21 days of culture in chondrogenic induction medium. All images are taken under 5 × magnifications. The size-bar on the images is equivalent to 250 µm

**Fig. 3:**
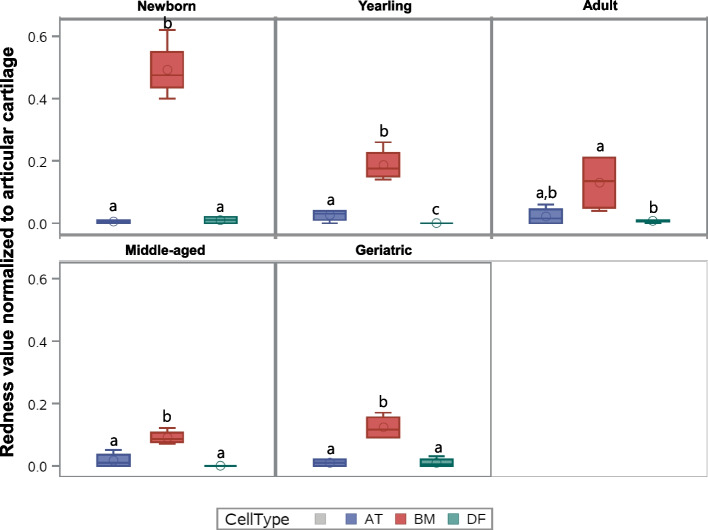
Proteoglycan Redness data of chondrogenic induced cells. Box plot showing proteoglycan Redness value of Safranin-O stained pellets of bone marrow (BM)- and adipose tissue (AT)- derived mesenchymal stromal cells and dermal fibroblasts (DF) (biological negative control) in five different age groups after normalization to articular cartilage (*n* = 4, *N* = 60). All pellets were cultured in chondrogenic induction medium for 21 days. Cell types within the same age group not labeled with the same letter are significantly different from each other (*p* < 0.05)

Control articular cartilage sections showed no significant difference between slide-position or batches. Figure 2 shows representative images of BM-MSC pellets, AT-MSC pellets, and DF pellets from horses in five different age groups after Safranin-O staining. None to minimal red staining was detectable in pellets made from AT-MSCs or DFs (biological negative control). Likewise, no redness was detected in the non-induced pellets or epidermal samples. When comparing cell types, BM-MSC pellets had a significantly higher proteoglycan content than pellets from AT-MSCs or DFs (*p* < 0.0001) (Figs. 2 and 3). The proteoglycan content was significantly higher in chondrogenic BM-MSC pellets compared to AT-MSC pellets in all age groups except adults. No difference was seen in proteoglycan content, when comparing chondrogenic induced AT-MSCs and DFs except for yearlings (Fig. 3).

When comparing age groups within one cell type, BM-MSC pellets from newborns had a significantly higher proteoglycan content compared to all other age groups (*p* < 0.0001), and yearlings had a significantly higher proteoglycan content compared to middle-aged horses (*p* = 0.02). For AT-MSC pellets, no statistical difference was seen in Redness values between any age groups.

### Osteogenesis

#### Alkaline phosphatase (ALP) activity

**Fig. 4:**
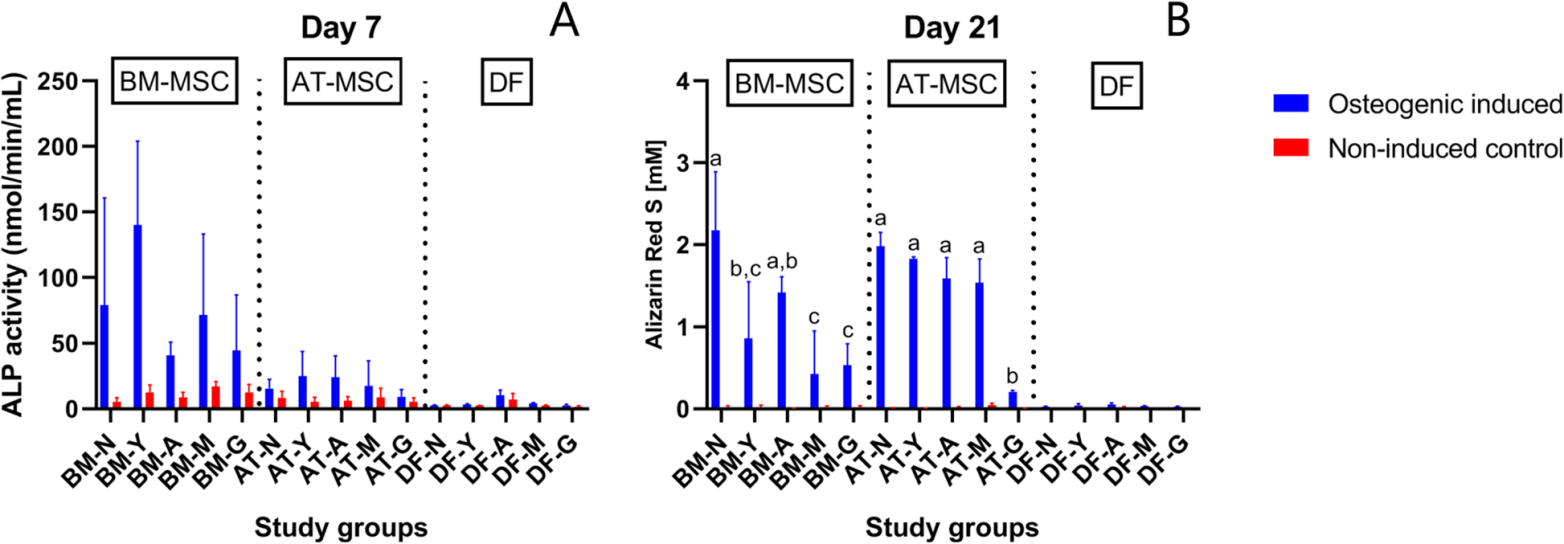
Alkaline phosphatase activity and Alizarin Red S concentration of osteogenic induced and control cells. Bar plot showing alkaline phosphatase (ALP) activity (**A**) and Alizarin Red S concentration (**B**) as a measurement of calcium deposits of equine bone marrow (BM)- and adipose tissue (AT)-derived mesenchymal stromal cells (MSC) and dermal fibroblasts (DF) (biological negative control) in five different age groups after respectively 7 or 21 days of culture in either osteogenic induction medium or expansion medium (non-induced control) (*n* = 4 horses per cell type per age group per medium). Age groups within the same osteogenic induced cell type not labeled with the same letter are significantly different from each other with regards to calcium deposition (*p* < 0.05). Error bars indicate standard deviation. ^†^N: Newborn, Y: Yearling, A: Adult, M: Middle-aged, G: Geriatric

There was a significant difference in ALP activity between osteogenic induced and non-induced BM-MSCs in newborn (*p* < 0.0001), yearling (*p* < 0.0001), and adult horses (*p* = 0.01) (Fig. 4). No difference was seen between osteogenic induced and non-induced cells in any of the AT-MSC age groups (Fig. 4). No statistical effect of donor age was seen in any of the pair-wise comparisons within cell type for BM-MSCs or AT-MSCs. Large inter-individual variation was seen within most age groups as indicated by the standard deviation (Fig. 4). When comparing cell types independent of age groups, ALP activity was seen in the following order: BM-MSC > AT-MSC > DF (*p* < 0.0001). When comparing cell types within age groups, BM-MSCs had a higher ALP activity compared to AT-MSCs in yearlings (*p* = 0.02).

#### Alizarin Red S (ARS) concentration

ARS concentration was significantly higher in osteogenic induced cultures compared to non-induced controls for all BM- and AT-MSC age groups, except middle-aged BM-MSC, and geriatric horses of both BM- and AT-MSCs. ARS concentration in osteogenic induced DFs (biological negative control) was significantly lower than in both BM- and AT-MSCs (*p* < 0.0001) (Fig. 4).

Calcium deposition decreased significantly with increasing donor age for both BM- and AT-MSCs (Fig. 4). For BM-MSCs, a significant difference was seen in pair-wise comparisons between newborn and yearlings (*p* = 0.0003). For AT-MSCs, a significant difference was only seen in pair-wise comparisons including geriatric horses (*p* < 0.0001).

When comparing cell types, the highest mean ARS value across all cell types was seen in BM-MSCs from newborn foals (mean ARS = 2.18 mM, SD = 0.72). No sigificant difference was detected between BM- and AT-MSCs in the newborn, adult, or geriatric age group. AT-MSCs had a significantly higher calcium deposition compared to BM-MSCs in yearling (*p* = 0.006) and middle-aged (*p* = 0.001) horses.

#### Gene expression—chondrogenesis

Five primer–probe sets (TNF, NODAL, MATN1, HAPLN1, and MEPE) had steady state mRNA expression below the assay detection level in almost all samples and were removed from the panel, resulting in a final panel of eighty-eight markers. Out of the eighty-eight biomarkers, eleven were affected by donor age in AT-MSCs, forty-five in BM-MSCs, and thirteen in DFs in chondrogenic induced pellets compared to monolayer control cells (Table 1). Nine biomarkers were affected by donor age in both BM- and AT-MSC pellets. Of these, six targets (PCNA, LOC100146270, BRCA1, BARD1, CASP8, DMP1) were up-regulated with increasing donor age in both BM- and AT-MSCs, whereas SMPD3 was down-regulated with increasing donor age in BM- and AT-MSCs. CLU and PDGFD were up-regulated in pellets from AT-MSCs and down-regulated in pellets from BM-MSCs with increasing donor age.

**Table 1:** Donor age affected genes within chondrogenic pellets

**CELL TYPE**	**GENE FUNCTION**			
	**Proliferation regulators**	**Signaling**	**Transcription factors**	**Extracellular matrix**
**AT-MSCs**	Up-regulated BARD1 (-) BRCA1^†^ (-) CASP8 (-) CLU ( +) LOC100146270 (-) PCNA ( +) PDGFD ( +)	Up-regulated MET		Up-regulated DMP1
				Down-regulated CRTAC1 SMDP3^†^
**BM-MSCs**	Up-regulated BARD1 (-) BRCA1^†^ (-) CASP8 (-) CCND1 ( +) CDKN1A (-) CTNNB1 ( +) GLB1^†^ (-) HDGF ( +) LOC100146270 (-) MYC ( +) PCNA ( +) PHB (-) TGFA ( +) TP53^†^ (-)	Up-regulated GDF6 PHEX	Up-regulated BMP4 BMP7 TGFB3	Up-regulated COL4A1 DMP1 LOC100146589
	Down-regulated BCL2 ( +) CLU ( +) IGF1^†^ ( +) PDGFD ( +)	Down-regulated ALPL^†^ ABI3BP FGFR3 IGFBP5 MIA^†^ S100A1 TNFRSF11B	Down-regulated BMP6 SNAI2^†^	Down-regulated ACAN CHADL COL1A1 COL9A2 COL11A1 COL12A1 COMP MMP13^†^ SMPD3^†^ SPARC
**DFs**	Down-regulated BCL2 ( +) CCND1 ( +) CTGF ( +) HGF ( +) TP53^†^ (-)	Down-regulated GDF6 TIMP2 SMOC1	Down-regulated GLI3 RUNX3	Down-regulated ACAN COL5A3 SMPD3^†^

All listed genes were significantly affected by donor age within pellets made from adipose tissue (AT)- and bone marrow (BM)-derived mesenchymal stromal cells (MSC) and dermal fibroblasts (DF) from horses in five different age groups (*n* = 4, *N* = 60) after 21 days of culture in chondrogenic induction medium as determined by one-way ANOVA when *p* < 0.05. Up-regulated genes indicate an up-regulation in gene expression with increasing donor age. Down-regulated genes indicate a down-regulation in gene expression with increasing donor age. ^†^: Categorized based on main function related to chondrogenesis, but could be categorized in more than one annotation category, ( +): Enhance proliferation, (-): Inhibits proliferation

Chondrogenic differentiation capacity as indicated by a general increase in expression of chondrogenic markers was more prominent in pellets from BM-MSCs (Additional file 1—heatmap). SOX5 and SOX9 are markers of early chondrogenesis. Expression of SOX5 was significantly higher in BM-MSC pellets in all age groups (*p* < 0.0001) and in AT-MSC pellets from newborn, yearling, and adult horses (*p* ≤ 0.003) compared to non-induced controls. Expression of SOX9 was significantly higher in BM-MSC pellets from newborns and yearlings (*p* ≤ 0.009) compared to non-induced controls, whereas no difference was seen for AT-MSCs or DFs compared to non-induced controls. CTGF and TGFB3 are signaling and transcription factors. For BM-MSCs, TGFB3 expression was significantly higher in pellets compared to non-induced controls in all age groups (*p* ≤ 0.002), whereas CTFG only showed difference in pellets versus non-induced controls in newborns, yearlings, and adults (*p* ≤ 0.003). There was no difference between pellets and non-induced controls from AT-MSCs or DFs. Expression of the specific marker of cartilage COL2A1 was higher in pellets from BM-MSCs (*p* < 0.0001) and AT-MSCs (*p* ≤ 0.0009) in all age groups compared to non-induced controls. Expression of essential cartilage extracellular matrix components COMP and ACAN were significantly higher in pellets than non-induced controls from both BM- and AT-MSCs in all age groups (*p* < 0.0001). For MIA, there was a greater expression in BM-MSC pellets compared to non-induced controls in all age groups (*p* < 0.0001), whereas no difference was seen in AT-MSCs or DFs. No difference was seen in expression of the fibrocartilage marker COL1A1 between pellets and non-induced cells in any of the cell types.

**Fig. 5:**
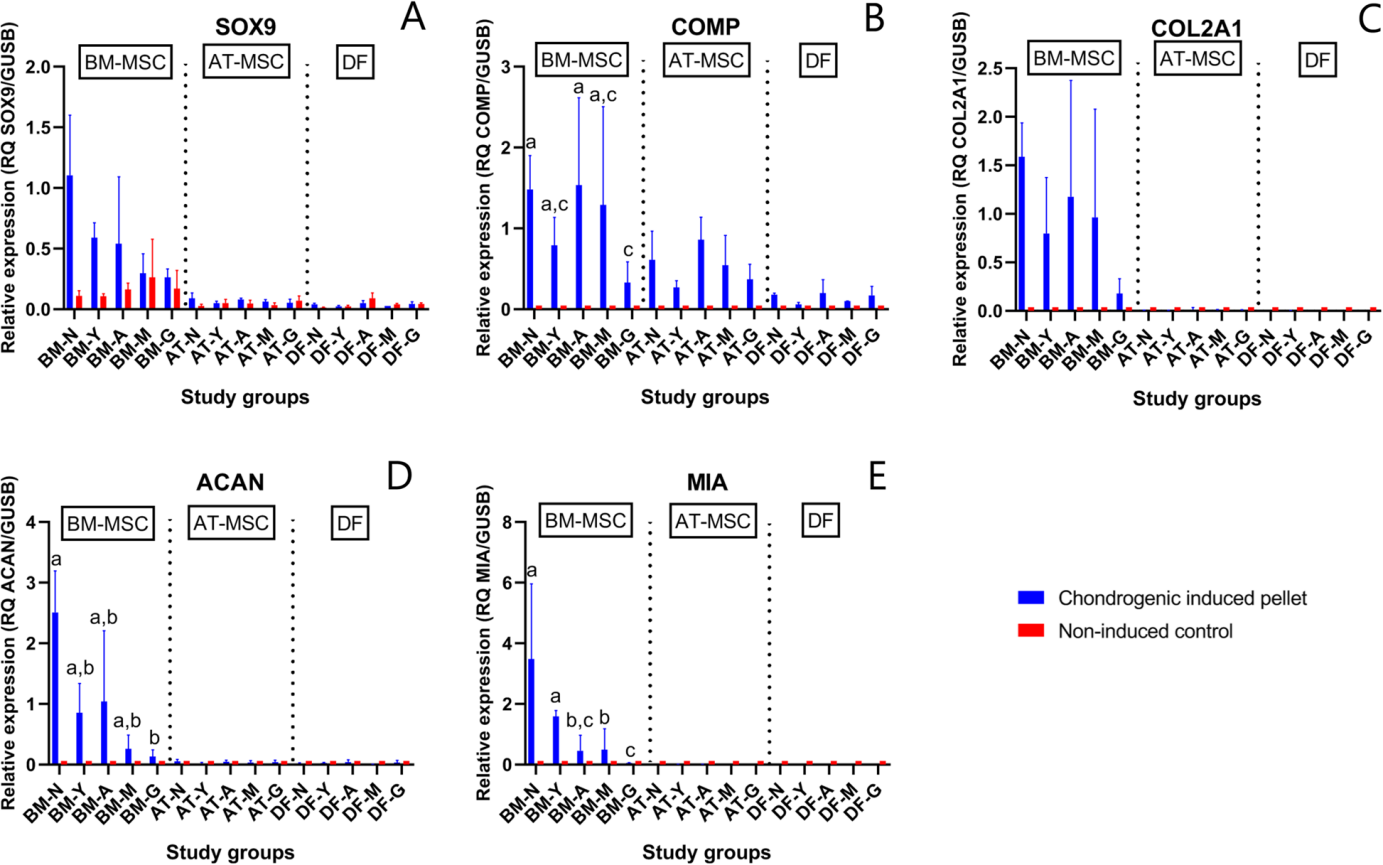
Gene expression of selected chondrogenic biomarkers. Bar plots with standard deviation illustrating mean relative gene expression of SRY-box transcription factor 9 (SOX9) (**A**), cartilage oligomeric matric protein (COMP) (**B**), collagen type 2 alpha 1 (COL2A1) (**C**), aggrecan core protein (ACAN) (**D**), and cartilage-derived retinoic acid-sensitive protein (MIA) (**E**) in chondrogenic induced pellets and non-induced monolayer controls from bone marrow (BM)- and adipose tissue (AT)-derived mesenchymal stromal cells (MSCs) and dermal fibroblasts (DF) from horses in five different age groups (*n* = 4 horses per age group per cell type per medium). Age groups within chondrogenic induced BM-MSCs not marked with the same letter are significantly different from each other (*p* < 0.05). No significant donor age effect was seen for SOX9 or COL2A1. Note different y-axes. ^†^N: Newborn, Y: Yearling, A: Adult, M: Middle-aged, G: Geriatric

When comparing the cell types independent of age group, BM-MSCs had a significantly greater expression of chondrogenic markers ACAN, COL2A1, MIA, and SOX9 than AT-MSCs or DFs (*p* < 0.0001). No difference was seen between AT-MSCs and DFs for the same biomarkers. Within BM-MSCs, expression of COMP and ACAN was significantly higher in pellets from newborn compared to geriatric horses (*p* = 0.01 and *p* = 0.001, respectively). Likewise, MIA was up-regulated in newborn BM-MSC pellets compared to all other age groups (*p* < 0.0001) except yearlings, who also had a higher expression compared to all older age groups (*p* ≤ 0.0006) (Fig. 5). Changes in COL2A1 were not significant. BM-MSC pellets from newborn foals had a significantly higher expression of COL11A1 compared to middle-aged (*p* = 0.03) and geriatric horses (*p* = 0.0003). Pellets from geriatric horses had a significantly higher expression of COL4A1 compared to newborn (*p* < 0.0001) and adult horses (*p* = 0.02). Pellets from middle-aged horses had significantly lower expression of COL1A1 compared to BM-MSC pellets from newborn (*p* = 0.01) and yearlings (*p* = 0.006), but no difference was found in pair-wise comparisons involving geriatric horses.

#### Gene expression—osteogenesis

Out of the eighty-eight biomarkers included in the final panel, fifteen were affected by donor age in AT-MSCs, thirty in BM-MSCs, and thirty-two in DFs when assessing osteogenic induced cells (Table 2). Seven of the targets were affected by donor age in both BM- and AT-MSCs. Of these, five targets (BGN, COL11A1, CTNNB1, HGF, SMOC) were up-regulated with increasing donor age in both BM- and AT-MSCs. CASP3 and CLU were up-regulated in osteogenic induced AT-MSCs and down-regulated in BM-MSCs with increasing donor age.

**Table 2:** Donor age affected genes within osteogenic monolayer cultures

**CELL TYPE**	**GENE FUNCTION**			
	**Proliferation regulators**	**Signaling**	**Transcription factors**	**Extracellular matrix**
**AT-MSCs**	Up-regulated CASP3 (-) CLU ( +)	Up-regulated CRYAB MET TNFRSF11B	Up-regulated SOX5	
	Down-regulated CTNNB1 ( +) HGF ( +)	Down-regulated SMOC^*†*^ TNFSF11	Down-regulated BMP2 BMP4	Down-regulated BGN COL11A1 SPARC
**BM-MSCs**	Down-regulated AQP1 ( +) BARD1 (-) CASP3 (-) CDKN1A (-) CLU ( +) CTNNB1 ( +) FGF5 ( +) FGF18 ( +) HDGF ( +) HGF ( +) IGF1^*†*^ ( +) MYC ( +) PDGFD ( +) TP53^*†*^ (-) VEGFA ( +)	Up-regulated SOST Down-regulated CAVIN1 GDF6 TIMP2 SMOC^*†*^	Down-regulated BMP6 GLI3 RUNX2 TGFB1	Up-regulated IBSP Down-regulated ACAN BGN COL11A1 DMP1 LOC100146589
**DFs**	Down-regulated BCL2 ( +) CASP3 (-) CDKN1A (-) FGF5 ( +) HGF ( +) PDGFD ( +) TP53^*†*^ (-) VEGFA ( +)	Up-regulated ALPL CRYAB EB2F S100A1 SOST TNFRSF11B	Up-regulated BMP6 SOX9 Down-regulated BMP2 BMP4 RUNX2 RUNX3 SP7 TGFB1 TGFB2	Down-regulated BGN COL1A1 COL4A1 COL11A1 COL12A1 FN1 LOC100146589 SPARC SPP1

All listed genes were significantly affected by donor age within monolayer cultures of adipose tissue (AT)- and bone marrow (BM)-derived mesenchymal stromal cells (MSC) and dermal fibroblasts (DF) from horses in five different age groups (*n* = 4, *N* = 60) after 21 days of culture in osteogenic induction medium as determined by one-way ANOVA when *p* < 0.05. Up-regulated genes indicate an up-regulation in gene expression with increasing donor age. Down-regulated genes indicate a down-regulation in gene expression with increasing donor age. ^†^: Categorized based on main function related to osteogenesis, but could be categorized in more than one annotation category, ( +): Enhance proliferation, (-): Inhibits proliferation

**Fig. 6:**
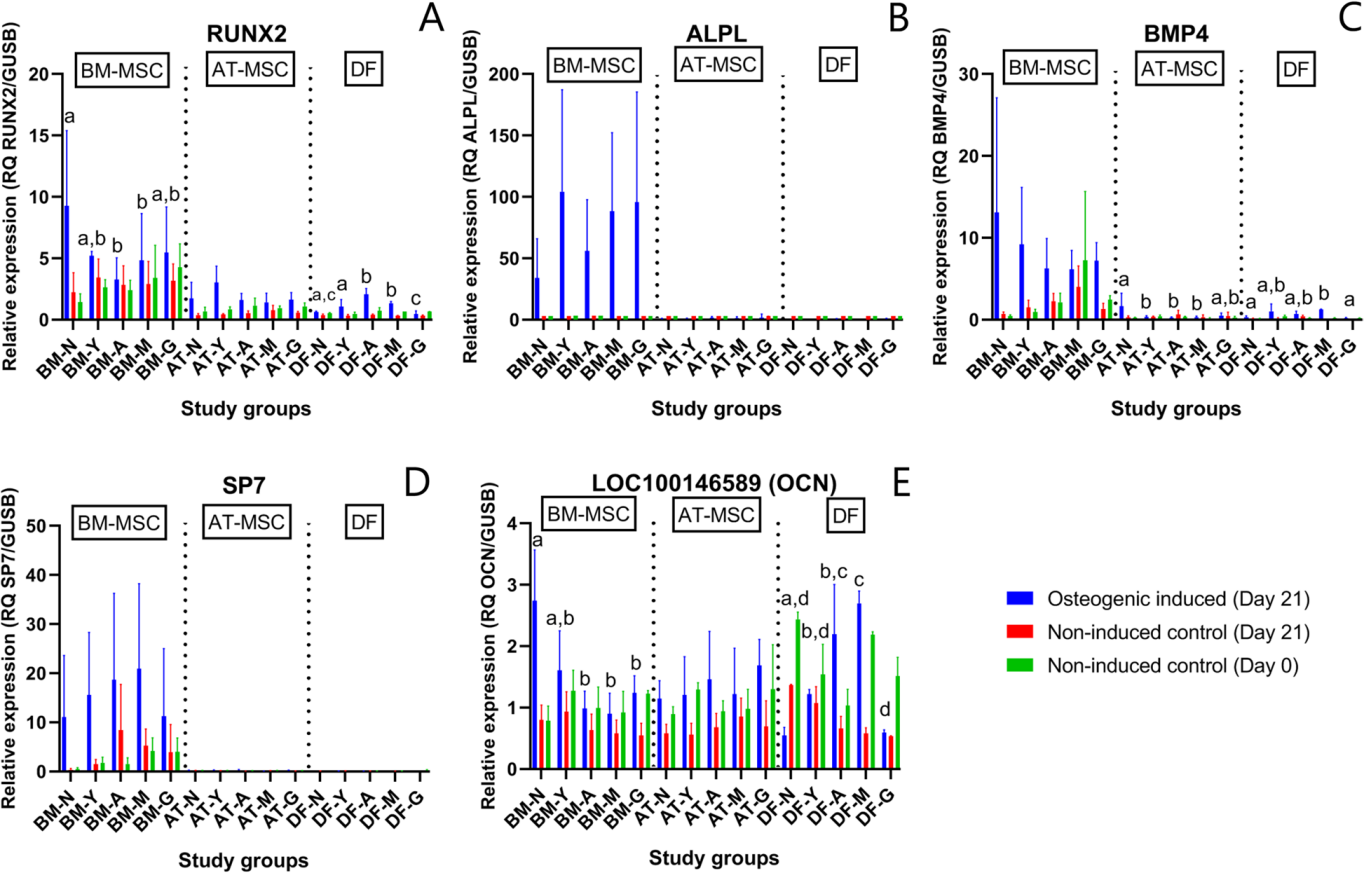
Gene expression of selected osteogenic biomarkers. Bar plots with standard deviation illustrating mean relative gene expression levels of runt-related transcription factor 2 (RUNX2) (**A**), alkaline phosphatase (ALPL) (**B**), bone morphogenic factor 4 (BMP4) (**C**), osterix (SP7) (**D**), and osteocalcin (LOC100146589) (**E**) in osteogenic induced cells after 21 days of induction and non-induced controls at day 0 and day 21. The cells consist of bone marrow (BM)- and adipose tissue (AT)-derived mesenchymal stromal cells (MSCs) and dermal fibroblasts (DF) from horses in five different age groups (*n* = 4 horses per age group per cell type per medium). Age groups within the same osteogenic induced cell type not marked with the same letter are significantly different from each other (*p* < 0.05). Note different y-axes. ^†^N: Newborn, Y: Yearling, A: Adult, M: Middle-aged, G: Geriatric

Increase in multiple osteogenic factors occurred in MSCs after culture in osteogenic induction medium for 21 days when compared to day 21 non-induced controls with the largest fold changes occurring in BM-MSCs (Fig. 6). Early marker of osteogenesis RUNX2 was significantly up-regulated in AT-MSCs from all age groups (*p* ≤ 0.001) and in BM-MSCs from newborn foals (*p* < 0.0001). Across all age groups, RUNX2 expression followed the pattern of; BM-MSCs > AT-MSCs > DFs (*p* < 0.0001). Another early marker of osteogenesis ALPL also showed significantly enhanced expression after osteogenic induction in BM-MSCs (*p* < 0.0001) and AT-MSCs (*p* ≤ 0.02) in all age groups when compared to non-induced cells on day 21. When comparing cell types, osteogenic induced BM-MSCs had significantly higher ALPL gene expression compared to AT-MSCs in all age groups (*p* ≤ 0.03). BMP4 was significantly up-regulated in osteogenic induced cells compared to non-induced day 21 cells for AT- and BM-MSCs in newborns (*p* = 0.005 and *p* < 0.0001, respectively), and in BM-MSCs in yearlings (*p* = 0.0004). Later markers of osteogenesis were also upregulated after osteogenic induction compared to non-induced cells on day 21. For osterix (SP7), enhanced expression was only observed in newborn (*p* = 0.002) and yearling BM-MSCs (*p* = 0.009). Across all age groups, BM-MSCs had a higher expression of SP7 compared to AT-MSCs and DFs. A significant difference in osteocalcin (LOC100146589) was also observed between osteogenic induced and non-induced cells at day 21 for AT-MSCs from newborns, yearlings, and adults (*p* ≤ 0.02) and in BM-MSCs from newborns (*p* < 0.0001). In newborns, expression of LOC100146589 was higher in osteogenic induced BM-MSCs than AT-MSCs (*p* = 0.04) and DFs (*p* < 0.0001). No difference was observed in LOC100146589 expression between BM- and AT-MSCs in any of the other age groups. When comparing all three osteogenic induced cell types independent of donor age, no difference was seen in LOC100146589 expression (*p* ≥ 0.2). COL1A1 expression was higher in non-induced compared to induced cells at day 21 for all three tissue types in all age groups.

When comparing within cell types, no effect of donor age was seen on ALPL expression in osteogenic induced BM- or AT-MSCs. The same was true for RUNX2 expression in AT-MSCs, but a significant decrease in RUNX2 expression was seen with increasing donor age in BM-MSCs, specifically between newborn and adults (*p* = 0.009), and newborn and middle-aged horses (*p* = 0.049). With regards to later markers of osteogenesis, SP7 and COL1A1, no effect of donor age was seen in osteogenic induced AT- or BM-MSCs. BM-MSCs from newborns had a significantly higher LOC100146589 expression compared to adult (*p* = 0.0006), middle-aged (*p* < 0.0001), and geriatric horses (*p* = 0.02) (Table 2 and Fig. 6).

## Discussion

Results from the present study support the hypothesis that increasing donor age is a major variable impacting equine BM- and AT-MSCs in vitro chondrogenic and osteogenic differentiation performance with decreasing capacities following non-linear kinetics, which is comparable to previous reports in other species [21–23, 25, 28–31]. Other research groups have reported no age related changes in MSC differentiation potential [32–34], however, their studies differed in culture conditions and lacked very young individuals in the study populations.

Of the targets affected by donor age in both AT- and BM-MSCs, none of the targets affected under chondrogenic culture were intersecting with targets affected under osteogenic culture. This suggests that the biomarkers affected by donor age in MSCs are influenced by the differentiation protocols, which is likely as different transcription factors and hence pathways are activated in the cells together with pelleting versus monolayer culture. In support of this finding, a recent proteomics study by Peffers et al*.* also reported no intersection of age-affected proteins in chondrogenic and osteogenic constructs made from human BM-MSCs [23]. The highest proportion of biomarkers affected by donor age was seen in BM-MSCs, which correlates with the finding that BM-MSCs also had the highest initial chondrogenic and osteogenic potential in newborns.

## Proliferation

As expected, some proliferation markers were down-regulated after inducing the cells to undergo chondrogenesis and osteogenesis. Of greater interest are, however, the six genes related to proliferation (except for DMP1) that were up-regulated with increasing donor age in both chondrogenic induced BM- and AT-MSCs, as they may be markers of genes consistently affected by donor age in equine MSCs under chondrogenic induction. Stenderup et al. reported that age related changes in bone formation were associated with lower MSC proliferative capacity and hence lower cell numbers rather than a lower function of the MSCs [34]. To control for this factor, all our assays were performed at passage 4 and all cells were plated at the same density in all steps. The tumor suppressors p16, BRCA1, and BARD1 and apoptosis factor CASP8 were all up-regulated in chondrogenic induced MSCs from aged donors. This may be related to cellular senescence or to various responses to the pelleting procedure as less oxygen is provided to the centrally located cells, which thereby would make them more prone to undergo cell death if not adapted to hypoxia as seen on the H&E slides [19, 35]. Increasing numbers of senescent cells have been reported in AT-MSCs from aged horses, together with a decline in cellular proliferation of BM- and AT-MSCs from aged horses, which is considered a feature of cellular senescence [36, 37]. As senescent cells have been shown to lose their differentiation capacity and to secrete inhibitory factors to surrounding cells [38], paracrine signaling would be a relevant factor to investigate in future studies, together with identification and localization of potentially senescent cells in the pellets.

## Donor age and chondrogenesis

Our data showed that BM-MSCs have higher potential for generating cartilage in vitro under standard chondrogenic induction conditions than AT-MSCs, which is supported by previous equine studies [14, 19, 39]. Interestingly, the chondrogenic differentiation performance of AT-MSCs very closely resembled that of DFs (biological negative control) in all age groups. AT-MSC pellets had a higher expression of COL1A1 and lower expression of COL2A1 compared to BM-MSCs. Expression of COL1A1 is documented in chondrogenic induced MSC pellets [12, 19], but is considered undesirable since COL1A1 is a component of biomechanically inferior fibrocartilage when compared to hyaline cartilage with high COL2A1 content [4]. In contrast, other studies have reported a chondrogenic differentiation potential of AT-MSCs, although lower than seen for BM-MSCs [14, 39]. The different results may be related to different culture conditions. Together, this highlights the limitations of AT-MSCs as a therapeutic choice for direct cartilage regeneration with regards to direct differentiation potential.

Despite the higher capacity for chondrogenesis in BM-MSCs, our data showed a significant decrease in proteoglycan content with increasing donor age in BM-MSC pellets already in pair-wise comparisons between newborn and yearlings. This finding on a protein level was supported by a decrease in ACAN gene expression with increasing donor age, as aggrecan is an essential proteoglycan in articular cartilage [4]. Surprisingly, the higher extracellular proteoglycan content in BM-MSCs from young horses was not reflected in pellet size, where no statistical difference was detected as a function of donor age. This may be partly due to a presumably increased number of senescent cells in BM-MSC pellets from old horses as GLB1 expression was up-regulated in aged horses, and senescent BM-MSCs are reported to have a larger cell morphology [40]. On a morphological level, BM-MSC pellets from newborn foals were most uniform in redness and showed characteristics representative of native articular cartilage with areas of lacunae occupied by single cells, proteoglycan rich matrix, and minimal necrotic cells. In the older age groups, more necrotic cells appeared centrally, which was also seen in pellets from AT-MSCs and DFs in all age groups. A zonal architecture of chondrogenic induced pellets have been reported previously [19, 41]. The lack of or minimal necrotic center in newborn BM-MSC pellets is particularly interesting as it suggests a cellular adaptation to lower oxygen levels. Such a characteristic would be an important feature, as articular chondrocytes live under hypoxic conditions with low oxygen levels between 1 and 10% depending on distance to the articular surface [9]. Extent of necrotic core as a function of donor age and cell type may also be caused by different diffusion efficiency of oxygen and nutrients in matrices of different pellet types, which is likely as pellets from aged BM-MSCs, AT-MSCs and DFs appeared denser with less extracellular matrix. Pellet size may also be a contributing factor, but no significant difference in pellet size was seen as a function of donor age in BM-MSCs, and the pellet size of newborn BM- and AT-MSCs were not significantly different from each other.

## Donor age and osteogenesis

Our data showed that BM-MSCs from young horses had higher in vitro potential for generating bone under standard osteogenic induction conditions than AT-MSCs, which is supported by previous human and equine studies [18, 20, 42–44]. On the other hand, Kang et al*.* reported that osteogenic induced canine AT-MSCs had higher ALP activity and mineralization compared to BM-MSCs after 14 days of culture, but that in vivo healing of bone radial defects was slightly better when using BM-MSCs [45]. Chung et al. demonstrated similar osteogenic differentiation potential between canine BM- and AT-MSCs, and found that hypoxia inhibited the proliferation of both cell types and osteogenic differentiation of AT-MSCs [46], which should be considered when using MSCs in hypoxic environments such as fractures and infections [47].

When including donor age as a factor, Asumda et al*.* reported that BM-MSCs from 15 months old rats were unable to perform chondrogenic or osteogenic differentiation [21]. This was partly supported by our data where only minimal chondrogenic and osteogenic differentiation was seen in BM-MSCs from geriatric horses with no statistical differences in ALP activity or calcium deposition between non-induced and induced cells. ALP is an early marker of osteogenesis and is highly expressed in mineralized tissue, where it enzymatically degrades inhibitory pyrophosphates that bind calcium to enhance mineralization, and ALP is considered a hallmark of osteogenic differentiated cells in culture with highest expression in equine MSCs 6 to 7 days after osteogenic induction [48, 49]. Interestingly, ALP activity was significantly higher in osteogenic induced cells versus non-induced cells in BM-MSCs from newborn, yearling, and adult horses, but AT-MSCs and DFs demonstrated no difference in ALP activity in any age group. On a gene level, our data showed the highest ALPL gene expression in BM-MSCs and no significant effect of donor age. Chen et al*.* [27] and Shi et al*.* [33] previously reported that human and mouse AT-MSCs osteogenic differentiation potential was less affected by donor age than BM-MSCs. This finding was partly supported by our data, where a decrease in ARS concentration of osteogenic induced BM-MSCs occurred already between newborn and yearlings, whereas calcium deposition only decreased in AT-MSCs from geriatric horses. Differences in initial calcium deposition may be a contributing factor, as the highest ARS concentration was reported in newborn BM-MSCs although not significantly different from newborn AT-MSCs. As mineralization depends on multiple factors [49], a decrease in ARS concentration with increasing donor age is possible even if no statistical donor age effect was seen for ALP.

The biggest fold changes after 21 days of osteogenic induction were seen in early markers of osteogenesis (e.g. RUNX2 and ALPL). RUNX2 is an early marker of osteogenesis and an essential transcription factor for activation of osteoblast associated genes like osteocalcin and ALPL [50]. Down-regulation of RUNX2 seen already in BM-MSCs from adult horses could be a major player in the decrease in osteogenic differentiation seen with increasing donor age in BM-MSCs, as its change in expression followed the pattern relatively well of downstream targets. Some of the later markers of osteogenesis including SP7 (osterix) and LOC100146589 (osteocalcin) were only significantly up-regulated after osteogenic induction in young horses. Curiously, osteocalcin was also found in day 0 DFs. Osteocalcin is specifically secreted by osteoblasts and is essential for mineralization. The low expression of osteocalcin may be partly explained by it mainly being produced by mature osteoblasts during bone mineralization [49]. Previous studies have shown higher expression levels after longer culture in osteogenic medium [48, 51]. Osteocalcin also plays an important role in the relationship between energy metabolism and bones, and are released from osteoblasts into the circulation where it targets other organs [49], which may explain its presence in DFs.

## Methodological considerations

All cells were collected immediately after euthanasia. This is not believed to affect the translational value of the results into autologous transplantations, as no differences have been reported in MSCs from newly euthanized and anesthetized horses [52]. The cells were collected from a mixture of female and male horses, with both genders represented in each age group. However, there was a higher presentation of cells from female horses, but in vitro mesodermal differentiation has been shown not to be affected by gender [53], and is as such not believed to bias the results of this study. Female BM-MSCs have, however, been reported to have increased expression of interferon-γ-receptor 1and interleukin-6-β (IL-6 β), and to be more potent in T cell proliferation suppression relevant for immunomodulation [53]. IL-6 has also been shown to enhance BMP2-induced osteoblastic differentiation of human BM-MSCs, but was not analyzed for gender in these studies [54, 55].

Chondrogenic differentiation of MSCs has been described using different induction mediums containing e.g. transforming growth factor β_3_ (TGF-β_3_) and BMP6 [56, 57]. Selection of chondrogenic induction medium containing TGF-β_1_ and dexamethasone for the present study was based on large, high-throughput mRNA profiling studies, showing this cocktail enhanced expression of hyaline articular cartilage markers, while downregulating unwanted pro-osteogenic markers [19, 58]. For assessment of chondrogenic differentiation on a transcriptional level, non-induced monolayer cells were used as a negative control. Inclusion of pellets kept under expansion medium for 21 days would have been an optimal negative control and was included in the original protocol. However, pellets from aged horses and DFs were not structurally stable when kept under those conditions for the experimental period.

It has previously been reported that DFs incapable of osteogenic differentiation can produce non-apatitic spontaneous calcium deposits when cultured with more than 2 mM β-glycerophosphate [59]. This was not demonstrated in our study, where DFs showed minimal calcium deposition after being cultured in osteogenic induction medium containing 10 mM β-glycerophosphate for 21 days. Likewise, DFs showed no chondrogenic differentiation after being cultured in chondrogenic induction medium for 21 days. The credibility of the differentiation results obtained from the MSCs is therefore strengthened, as the induction mediums were not able to produce measurable changes in DFs.

The present study investigated differentiation performance in culture. For equine clinical applications, further experiments that extend the assessment to cells in vivo and their potential to heal cartilage and bone injuries as a function of donor age will be valuable.

## Conclusions

The chondrogenic and osteogenic differentiation performance of equine BM- and AT-MSCs declined with increasing donor age. BM-MSCs from newborn foals had the highest in vitro chondrogenic and osteogenic potential compared to older age groups and AT-MSCs. Decrease in chondrogenic proteoglycan content and osteogenic calcium deposition was seen already in pair-wise comparisons between newborn and yearling BM-MSCs. AT-MSCs showed minimal ability to perform chondrogenic differentiation, but were less affected by donor age than BM-MSCs when making calcium deposits during osteogenesis, where no difference was seen except in geriatric horses. Gene expression analysis showed changes in regulators of proliferation and chondrogenic- and osteogenic differentiation markers with increasing donor age. Together, this highlights the importance of donor age considerations and MSC tissue type selection for MSC treatments in orthopedic conditions. Changes in differentiation potential in vitro, however, need to be held together with their in vivo potential to heal cartilage and bone injuries, which remains to be investigated as a function of donor age in horses.

## Methods

### Experimental samples

BM-MSCs, AT-MSCs, and DFs (biological negative control) were harvested immediately post-mortem from horses of mixed breeds across five different age groups. Four horses were used for each cell type and age group. Age groups were newborn (0 days old), yearling (15–17 months), adult (5–8 year old), middle-aged (12–18 year old), and geriatric (≥ 22 year old). All horses were euthanized due to reasons unrelated to this study. Experiments were performed at two different universities. An inter-assay control was applied and showed no effect of site. Plasticware, reagents, cell culture media, commercially available kits, and protocols were kept constant between sites and throughout the entire study.

### Bone marrow-derived MSC collection and isolation

BM was collected immediately post-mortem from the sternum of twenty horses (twelve female and eight male) with the following age distribution (mean (range)); newborn (0 days (0–0 days)), yearling (15.7 months (15.3–16 months)), adult (6.8 years (5–8 years)), middle-aged (15.5 years (13–18 years)), and geriatric (25.8 years (22–31 years)) (*n* = 4 horses per age group). For newborns, BM was collected as described by Vidal et al*.* [15] with a few modifications. Briefly, marrow trabecular bone was collected from the 4^th^-6^th^ sternebrae by sterile curettage after splitting the sternum along the midline, transported on ice to the laboratory in ice cold sterile Dulbecco’s phosphate buffered saline (dPBS – Thermo Fisher Scientific, Waltham, MA, USA) with 2% (v/v) amphotericin B (Thermo Fisher Scientific, Waltham, MA, USA), and 2% (v/v) penicillin/streptomycin (P/S – Thermo Fisher Scientific, Waltham, MA, USA) (isolation solution) and processed within 2 h of collection. Marrow trabecular bone was rinsed twice in 37 °C isolation solution, crushed, and grown as explant cultures in T75 flasks (Cellstar BioGreiner – VWR, Radnor, PA, USA) in Dulbecco’s modified Eagle’s medium (DMEM, 1 g/L glucose, with phenol red, GlutaMAX, and pyruvate – Thermo Fisher Scientific, Waltham, MA, USA), 10% (v/v) fetal bovine serum (FBS), 1% (v/v) P/S, and 1% (v/v) amphotericin B (isolation medium). After 48 h, BM crusts, non-adherent cells, and isolation medium were removed, and expansion medium was added containing DMEM (1 g/L glucose), 10% (v/v) FBS, and 1% (v/v) P/S. For the four older age groups, 20 mL BM aspirate was collected from the 4^th^-6^th^ sternebrae with a Jamshidi® bone biopsy needle (Henry Schein Vet, Dublin, OH, USA), and BM-MSCs were isolated using Ficoll-Paque® PREMIUM (GE Healthcare, Chicago, IL, USA) as previously described [15, 60]. Isolation medium was replaced with expansion medium 48 h after isolation.

### Adipose tissue-derived MSC collection and isolation

AT was collected from the gluteal region next to the tail base of twenty horses (ten female and ten male) as previously described [60] with the following age distribution (mean (range)); newborn (0 days (0–0 days)), yearling (15.8 months (15.3–16.1 months)), adult (6.8 years (5–8 years)), middle-aged (14.5 years (13–16 years)), geriatric (25.8 years (22–31 years)) (*n* = 4 horses per age group). Briefly, the gluteal area was surgically clipped and prepared, and 10 g of AT was collected through a ~ 8 × 8 cm surgical window. AT was transferred to a 50 mL falcon tube with ice cold isolation solution, transported on ice to the laboratory, and processed within 2 h of collection. AT was washed twice in isolation solution, dissected into smaller pieces, and visible blood vessels removed. AT was digested in sterile filtered (0.2 µm) enzyme medium consisting of DMEM (1 g/L glucose), 1% (v/v) P/S, 50 µg/mL gentamycin (Sigma-Aldrich, St. Louis, MI, USA), and 1 mg/mL collagenase type I (Worthington Biochemical Corp., Lakewood, NJ, USA) for 3 h at 37 °C and 30 rpm. Released cells were strained through a 70 µm cell strainer, washed twice in sterile dPBS, and centrifuged at 500 g for 5 min between washes. The pellet was then re-suspended in isolation medium supplemented with 50 µg/mL gentamycin and distributed into two T75 flasks. Medium was changed to expansion medium 48 h after isolation.

### Dermal fibroblast collection and isolation

DFs were collected from the gluteal region next to the tail base of twenty horses (fourteen female and six male) as previously described [19] with the following age distribution (mean (range)); newborn (0 days (0–0 days)), yearling (15.6 months (15.3–16 months)), adult (6 year (5–7 years)), middle-aged (14.5 years (12–18 years)), geriatric (27.5 years (22–32 years)) (*n* = 4 horses per age group). Briefly, 6 g of dermal tissue was harvested, transferred to a 50 mL falcon tube with ice cold isolation solution and transported on ice to the laboratory, where the tissue was further processed within 2 h of collection. Dermal tissue was washed twice in isolation solution, dissected into smaller pieces, and digested in sterile filtered (0.2 µm) enzyme medium consisting of dPBS, 1% (v/v) P/S, 1% (w/v) bovine serum albumin (BSA – Sigma-Aldrich, St. Louis, MI, USA), 50 µg/mL gentamycin, and 1 mg/mL collagenase type I for 2 h at 37 °C and 30 rpm. Released cells were filtered through a 70 µm cell strainer, washed twice in sterile dPBS, and centrifuged at 1000 g for 4 min between washes. The pellet was re-suspended in isolation medium supplemented with 50 µg/mL gentamycin, distributed into two T75 flasks and cultured at 37 °C in air with 5% CO_2_. Medium was change to expansion medium 48 h after isolation.

### Cell expansion and storage

Expansion medium was changed every 2–3 days. At approximately 80% confluence, plastic-adherent cells were passaged using Trypsin/EDTA (0.25%) (Thermo Fisher Scientific, Waltham, MA, USA), counted, and replated at a seeding density of 500,000 cells per T75 flask. At passage 2, cells were cryopreserved in Recovery-Cell Culture Freezing Medium® (Thermo Fisher Scientific, Waltham, MA, USA) at a concentration of 2–3 × 10^6^ cells/mL freezing medium in cryogenic vials (Thermo Fisher Scientific, Waltham, MA, USA). For chondrogenic and osteogenic assays, cells were thawed in 37 °C expansion medium and washed three times in dPBS before being plated and cultured in expansion medium at 37 °C and 5% CO_2_. Medium was changed every 2–3 days. Chondrogenic and osteogenic differentiation assays were performed at passage 4*.*

### Chondrogenic assays

#### Pelleting and chondrogenic differentiation

Passage 4 cells were suspended in chondrogenic induction medium consisting of DMEM (4.5 g/L glucose – Thermo Fisher Scientific, Waltham, MA, USA), 1% (v/v) P/S, 12.5 mg/mL BSA, 1 × insulin-transferrin-selenium-sodium pyruvate growth supplement (Thermo Fisher Scientific, Waltham, MA, USA), 1 × MEM non-essential amino acids (Thermo Fisher Scientific, Waltham, MA, USA), 100 nM dexamethasone (Sigma-Aldrich, St. Louis, MI, USA), 50 µg/mL L-ascorbic acid-2-phosphate sesquimagnesium salt hydrate (A2P – Sigma-Aldrich, St. Louis, MI, USA), and 10 ng/mL human recombinant transforming growth factor β_1_ (rhTGFβ_1_ – Sigma-Aldrich, St. Louis, MI, USA) at 500,000 cells per mL as described previously [19, 41, 58]. Pellets were generated by centrifuging 1 mL of cell suspension at 500 g for 3 min in a vented 1.5 mL polypropylene microcentrifuge tube. A total of fifteen pellets were made per cell line per biological replicate. After 24 h, pellets were transferred to a 1% (w/v) poly 2-hydroxyethyl methacrylate (polyHEMA – Sigma-Aldrich, St. Louis, MI, USA) coated 24-well plate with one pellet per well to enable suspension-culture. Pellets were cultured in freshly prepared chondrogenic induction medium for 21 days with 1 mL of complete medium change every 3 days. Passage 4 control cells from each cell line and biological replicate were seeded at 500,000 cells in one T75 flask and cultured as monolayer in expansion medium. Six pellets from yearling BM-MSCs were cultured in chondrogenic medium without rhTGF-β_1_ and dexamethasone as a non-induced control for pellet size and proteoglycan staining.

#### Chondrogenic pellet size

Out of the fifteen pellets per biological replicate, six were randomly chosen to be measured in size and assessed histologically. Pellets were gently washed with dPBS and transferred to a 24-well plate with one pellet and 0.5 mL dPBS per well. Standardized 2D brightfield images of the pellets were captured at 4 × magnification using a light microscope. The shortest and longest side of the pellet was measured in µm using Nikon NIS Analysis Software 4.13 (Nikon Instruments Inc., Melville, NY, USA). The measurements were averaged per pellet and per biological replicate.

#### Histological assessments of pellets

Six randomly-selected pellets per biological replicate were fixed in 4% paraformaldehyde at 4 °C for 24 h, and pre-embedded with two pellets per block in 2% (w/v) bacto™ agar (Thermo Fisher Scientific, Waltham, MA, USA) /2.5% (w/v) gelatin blocks as previously described to prevent folding at sectioning [61]. Next, the blocks were fixed in 4% paraformaldehyde for 24 h at 4 °C, before being placed in 70% ethanol at 4 °C for batched histological processing and paraffinization. Pellets were sectioned at 5 µm at approximate midline and stained with either Hematoxylin and Eosin (H&E) for cell morphology and pellet architecture or Safranin-O to evaluate proteoglycan content and distribution.

#### Proteoglycan staining and quantification

For Safranin-O staining, all sections were first stained with Weigert’s Iron Hematoxylin for 7 min to stain cell nuclei, and 1% (w/v) Fast Green (Sigma-Aldrich, St. Louis, MI, USA) for 5 min as a background stain. Proteoglycan sulfation was identified with a 0.05% (w/v) Safranin-O (Sigma-Aldrich, St. Louis, MI, USA) staining for 2 min as described previously [19]. The sections were stained on the same day in 30-slide racks in the same batch of Safranin-O and Fast Green stain, with refreshing of stain solution between every two racks. Mature equine articular cartilage was included as a positive control calibrator sample with three randomly placed slides in each rack. Non-induced BM-MSC pellets from a yearling and epithelial tissue from a foal were incorporated as negative controls with one slide of each per rack. Mounted sections were imaged in brightfield with a Zeizz AX10 Imager A2 microscope (Zeiss, Oberkochen, Germany) at 5 × magnification using the same microscope brightness and exposure settings for all images. Images of the 0.05% Safranin-O staining used in the experiment were captured using a chamber slide as a pure red calibrator. Redness values of each pellet were measured using Fiji software in ImageJ (https://imagej.net/Fiji/Downloads) as previously described [19], recorded and normalized to articular cartilage control sections, and averaged per biological replicate.

### Osteogenic assays

#### Osteogenic differentiation

Passage 4 cells were seeded as monolayer cultures at a density of 25,000 cells per well in 6-well plates (Thermo Fisher Scientific, Waltham, Ma, USA). Initially, the cells were cultured in expansion medium at 37 °C and 5% CO_2_ with media change every 2–3 days. Upon ~ 90% confluence, expansion medium was changed to osteogenic induction medium in three of the wells, while the other three wells were kept in expansion medium as a non-induced control. Osteogenic induction medium consisted of DMEM (1 g/L glucose), 10% (v/v) FBS, 1% (v/v) P/S, 100 nM dexamethasone, 0.05 mM ascorbic acid (A2P), and 10 mM β-glycerophosphate disodium salt hydrate (Sigma-Aldrich, St. Louis, MI, USA) as previously described [15, 48, 62]. Medium in both groups was changed every 3 days with 2 mL medium per well.

#### Alkaline phosphatase activity assay

After 7 days of osteogenic induction, alkaline phosphatase (ALP) activity was measured using a commercially available colorimetric kit (Alkaline Phosphatase Assay Kit – Abcam, Cambridge, UK) according to the manufacturer’s instructions and as described previously [63]. Briefly, the cells were washed with dPBS and harvested by Trypsin/EDTA (0.05%) (Thermo Fisher Scientific, Waltham, MA, USA) and cell scraping. The cell suspension from each well was transferred individually to a microcentrifuge tube, spun down, and washed in dPBS. The pellet was resuspended in 4 °C dPBS and centrifuged, supernatant discarded and 200 µL assay buffer was added to each pellet before homogenization in a QIAshredder (Qiagen, Germantown, MD, USA) at 20,000 g for 2 min, followed by top speed (21,000 g) for 15 min at 4 °C. Samples were transferred to cryogenic vials and stored at -80 °C until batch ALP assays were performed. ALP activity of the cells was measured in flat-bottomed 96-well plates by adding 5 mM p-nitrophenyl phosphate substrate and incubating for 60 min at 25 °C protected from light. Stop solution was added and optical density was measured at 405 nm using a microplate reader (Bio-Rad, Hercules, CA, USA). ALP activity was calculated based on standard curve solutions incorporated in the assay after subtracting mean blank values from all samples. All technical replicates were tested in duplicate with three osteogenic-induced and three non-induced wells per cell line. ALP activity levels of the technical replicates were averaged per biological replicate per cell type per medium.

#### Alizarin Red S assay

After 21 days of osteogenic induction, calcium deposition was measured with a commercially available colorimetric Alizarin Red S (ARS) quantification kit (ARed-Q – ScienCell, Carlsbad, CA, USA) following the manufacturer’s protocol based on a study by Gregory et al*.* [64]. In short, the osteogenic medium was removed and the cells were gently washed three times in dPBS without calcium and magnesium (Thermo Fisher Scientific, Waltham, MA, USA) before being fixed in 4% paraformaldehyde. Calcium deposits were then visualized by adding 40 mM ARS staining and incubating for 20 min with gentle shaking. The dye was removed and the wells were washed five times with milli-Q water with 5 min incubation between washes. Calcified minerals were extracted at low pH using 10% acetic acid and a cell scraper, followed by a heating process in parafilm-coated microcentrifuge tubes at 85 °C for 10 min and a cooling process on ice for 5 min. The solution was then neutralized with 10% ammonium hydroxide (pH between 4.1–4.5). ARS concentration of all samples was quantified by colorimetric detection at 405 nm in flat-bottomed 96-well plates using a microplate reader (Bio-Rad, Hercules, CA, USA) and calculated based on a standard curve incorporated in the assay after mean blank value subtraction. All technical replicates were analyzed in triplicates with three osteogenic-induced and three non-induced wells per cell line. ARS concentration was averaged per cell line per biological replicate per medium.

### Gene expression assays

#### RNA isolation and reverse transcription

After 21 days, chondrogenic and osteogenic cultures were harvested and processed for total RNA isolation. Chondrogenic pellets (nine from each cell line) were washed with dPBS and snap frozen in aliquots with three pellets per aliquot. Passage 4 control cells from monolayer cultures at approximately 80% confluence were extracted with QIAzol® (Qiagen, Germantown, MD, USA) and snap frozen in liquid nitrogen. Osteogenic cultures and non-induced day 0 and day 21 controls were harvested with QIAzol® and snap frozen. All samples were stored at -80 °C prior to RNA isolation. Monolayers and pellets were homogenized in 1 mL QIAzol® using a PowerGen 125 homogenizer (Thermo Fisher Scientific, Waltham, MA, USA). Pellets (three per aliquot) were homogenized three times for 30 s at high speed with a 2 min cooling on ice after each homogenization. Monolayers were homogenized twice. Additional 1 mL of QIAzol® was added and the sample was incubated for 5 min at room temperature before proceeding directly to RNA isolation. Total RNA was isolated using a spin column-based Qiagen RNeasy Mini Kit® (Qiagen, Germantown, MD, USA) with modifications as previously described [65], with pooling of technical replicates. A final ethanol precipitation was applied to the samples to remove processing contaminants. Quantification of purified RNA was performed using a Qubit BR Assay (Thermo Fisher Scientific, Waltham, MA, USA) and NanoDrop ND 1000 spectrophotometer (Thermo Fisher Scientific, Waltham, MA, USA). Quality of purified RNA was assessed with a Bioanalyzer 2100 (Eukaryotic Total RNA Nano & Pico Series II – Agilent Technologies, Santa Clara, CA, USA). All purified RNA samples met the following quality thresholds; 260/280 ratios of 1.7–2.1, 260/230 ratios of 1.8–2.28, and an Agilent RNA integrity number (RIN) of ≥ 7.4, with the exception of three samples (260/280 ratios of 1.60, 2.20, and 2.30) located in different groups (all three samples behaved as expected in down-stream analyses and thus were not excluded from the study). Removal of any genomic DNA contamination and the reverse transcription of total RNA to cDNA were performed using a commercially available kit as per manufacturer’s protocol (Maxima First Strand cDNA Synthesis Kit® for RT-qPCR with dsDNase – Thermo Fisher Scientific, Waltham, MA, USA). cDNA samples were diluted with nuclease-free water to 13.9 ng/uL and stored at -80 °C.

#### Real-time quantitative PCR

Commercially available, validated equine-specific TaqMan® primer–probe sets (FAM dye-labeled MGB probes—Thermo Fisher Scientific, Waltham, MA, USA) were used to quantitate steady state mRNA levels of ninety-three biomarkers selected based on functional annotation having biological relevance for cell proliferation, chondrogenesis, osteogenesis, or evidence in the literature of age-dependent variation. Details on the primer–probe sets are listed in Table 3. A robotic ViiA™ RT-qPCR System (Thermo Fisher Scientific, Waltham, MA, USA) was used to test the functionality of all primer–probe sets against an equine positive control sample consisting of mixed cDNA from a forty-three-sample pool of various tissues [66], day 35 whole fetus, and neonatal epiphyseal cartilage. Negative controls of RNase-free water and minus-template were incorporated, and each sample was run in duplicate. LinRegPCR was used to measure reaction amplification efficiencies and cycle threshold (Ct) values were calculated [67]. All targets showed amplification efficiencies close to 2, except for the negative controls that showed no amplification. Three commercially available equine-specific endogenous control TaqMan® primer–probe sets were tested in the same system against all experimental samples; β-2-microglobulin (B2M), β-glucoronidase (GUSB), and ribosomal protein lateral stalk subunit P0 (RPLP0). Using NormFinder software [68], GUSB was determined as having the most uniform performance across all cell types and age groups.

**Table 3:** Overview of TaqMan primer–probe sets used in RT-qPCR reactions

Gene ID	Gene name(s)	ThermoFisher Assay ID
ABI3BP	ABI family member 3 binding protein	Ec06625599_m1
ACAN	Aggrecan core protein	Ec03469667_m1
ALPL	Alkaline phosphatase	Ec06625502_g1
AQP1	Aquaporin	Ec06625425_m1
AREG	Amphiregulin	Ec06992855_m1
BARD1	BRCA1-associated RING domain 1	Ec07061151_m1
BAX	BCL2 associated X apoptosis regulator	Ec07016716_s1
BCL2	B-cell lymphoma 2	Ec07005800_g1
BGN	Biglycan	Ec03467971_m1
BMP2	Bone morphogenic factor 2	Ec06974239_m1
BMP3	Bone morphogenic factor 3	Ec07037656_m1
BMP4	Bone morphogenic factor 4	Ec03470252_s1
BMP6	Bone morphogenic factor 6	Ec03469925_m1
BMP7	Bone morphogenic factor 7	Ec04320876_m1
BRCA1	Breast cancer type 1	Ec07017862_s1
CASP3	Caspase 3	Ec03470391_m1
CASP8	Caspase 8	Ec06959413_m1
CAVIN1	Caveolae associated protein 1	Ec07036873_m1
CCND1	Cyclin D1	Ec07036996_m1
CDKN1A	p21, Cyclin-dependent kinase inhibitor 1A	Ec06955195_m1
CHADL	Chondroadherin	Ec03470206_m1
CLU	Clusterin	Ec03468575_m1
COL1A1	Collagen type 1 alpha 1	Ec03469676_m1
COL2A1	Collagen type 2 alpha 1	Ec03467411_m1
COL4A1	Collagen type 4 alpha 1	Ec06943950_m1
COL5A3	Collagen type 5 alpha 3	Ec06999559_g1
COL9A2	Collagen type 9 alpha 2	Ec03470083_m1
COL11A1	Collagen type 11 alpha 1	Ec07051918_m1
COL12A1	Collagen type 12 alpha 1	Ec03469523_m1
COMP	Cartilage oligomeric matrix protein	Ec03468062_m1
CRTAC1	Cartilage acidic protein –CEP-68	Ec07040335_g1
CRYAB	Crystalline-alpha B	Ec06997901_m1
CSF2	Colony stimulating factor 2	Ec03468208_m1
CTGF	Connective tissue growth factor	Ec06625777_gH
CTNNB1	Beta-catenin	Ec00991819_m1
DCN	Decorin	Ec03468474_m1
DMP1	Dentin matrix acidic phosphoprotein 1	Ec06992382_m1
EBF2	Early B-cell factor 2	Ec06966528_m1
EPGN	Epithelial mitogen	Ec06992859_m1
FGF1	Fibroblast growth factor 1	Ec01092738_m1
FGF5	Fibroblast growth factor 5	Ec04656774_m1
FGF18	Fibroblast growth factor 18	Ec03248217_g1
FGFR3	Fibroblast growth factor receptor 3	Ec03470545_m1
FN1	Fibronectin 1	Ec03470760_m1
GDF6	Growth differentiation factor 6	Ec07097112_m1
GLB1	Beta-galactosidase	Ec06954363_m1
GLI3	GLI family zinc finger 3	Ec06625512_m1
HAPLN1	Hyaluronan and proteoglycan link protein 1	Ec03468716_m1
HDGF	Hepatoma-derived growth factor	Ec07037751_m1
HGF	Hepatocyte growth factor	Ec07000054_m1
IBSP	Integrin Binding Sialoprotein	Ec06625402_m1
IGF1	Insulin-like growth factor 1	Ec03468689_m1
IGFBP5	Insulin-like growth factor binding protein 5	Ec03470296_m1
LOC100146-270	p16, Cyclin-dependent kinase 4 inhibitor B	Ec07037471_mH
LOC100146-589	Osteocalcin	Ec07103628_m1
MATN1	Matrilin-1	Ec06963427_m1
MEPE	Matrix extracellular phosphoglycoprotein	Ec06992377_m1
MET	MET proto-oncogene, receptor tyrosine kinase	Ec02622441_m1
MIA	Cartilage-derived retinoic acid-sensitive protein	Ec03469434_m1
MMP3	Matrix metalloproteinase 3	Ec03468676_m1
MMP13	Matrix metalloproteinase 13	Ec03467796_m1
MYC	c-myc	Ec07007511_m1
NODAL	Nodal growth differentiation factor	Ec07036659_m1
OMD	Osteomodulin	Ec06625496_m1
PCNA	Proliferating cell nuclear antigen	Ec06974312_m1
PDGFD	Platelet-derived growth factor subunit D	Ec06997714_m1
PHB	Prohibitin	Ec07055990_m1
PHEX	Phosphate regulating endopeptidase homolog, X-linked	Ec07034292_s1
PTCH2	Patched 2	Ec06625424_g1
RUNX2	Runt-related transcription factor 2	Ec03469741_m1
RUNX3	Runt-related transcription factor 3	Ec06625430_g1
S100A1	S100 calcium binding protein A1	Ec03470173_g1
SMOC1	SPARC related modular calcium binding	Ec06978758_m1
SMPD3	Sphingomyelin phosphodiesterase 3	Ec06625668_m1
SNAI2	Snail family transcriptional repressor 2	Ec06625397_m1
SOST	Scherostin	Ec07036868_m1
SOX5	SRY-box transcription factor 5	Ec01552798_m1
SOX9	SRY-box transcription factor 9	Ec03469763_s1
SP7	Osterix	Ec06625770_g1
SPARC	Osteonectin	Ec03818202_m1
SPP1	Osteopontin, Secreted phosphoprotein 1	Ec06992376_g1
TBX3	T-box 3	Ec07003300_m1
TERT	Telomerase reverse transcriptase	Ec06972692_m1
TGFA	Transforming growth factor alpha	Ec06949183_m1
TGFB1	Transforming growth factor beta 1	Ec06625477_m1
TGFB2	Transforming growth factor beta 2	Ec07074189_g1
TGFB3	Transforming growth factor beta 3	Ec00682163_m1
TIMP2	Metallopeptidase inhibitor 2	Ec03470558_m1
TNF	Tumor necrosis factor	Ec03467871_m1
TNFRSF11B	Osteoprotegerin	Ec07007303_m1
TNFSF11	RANKL, TNF superfamily member 11	Ec06625532_m1
TP53	Tumor protein 53	Ec03470648_m1
VEGFA	Vascular endothelial growth factor A	Ec03467879_m1
**B2M**	Beta-2-microblobulin	Ec03468699_m1
**GUSB**	Beta-glucoronidase	Ec03470630_m1
**RPLP0**	Ribosomal protein lateral stalk subunit P0	Ec04947733_g1

Gene expression in the experimental samples was subsequently analyzed by RT-qPCR using the BIOMARK HD System (Fluidigm Corp., South San Francisco, CA, USA) as previously described [69] including positive and negative controls as detailed above. The Fluidigm assay was carried out using 96.96 dynamic arrays (Fluidigm Corporation, South San Francisco, CA, USA) according to manufacturer’s instructions. Data were analyzed with Fluidigm Real-Time PCR Analysis Software in the BIOMARK instrument, where Ct values were calculated. Delta Ct values were determined for each sample by subtracting the corresponding Ct value of the endogenous control (GUSB). The positive control was used as a calibrator to calculate ΔΔCt values. Relative expression (RQ) of the gene targets were calculated using the 2^−ΔΔCt^ method [70]. RQ levels were used for graphical bar/box-plot presentations made in GraphPad Prism 8.0.1 (GraphPad Prism, San Diego, CA, USA) or SAS 9.4 (SAS Institute Inc., Cary, NC, USA) and Ln(RQ) values were used for heatmap and statistical analysis. Gene expression heatmap visualization was done on averaged Ln(RQ) levels for each study group, grouping the samples according to cell type, donor age, and medium, and genes according to biological annotation. A heatmap was generated in RStudio 3.6.0 (R, Vienna, Austria) using the *heatmap ggplot* function.

### Statistical analysis

Sample size was determined by two-sample t-test power analysis in R (version 3.6.0) using BM- and AT-MSC calcium deposition pilot data (not shown) from newborn and geriatric horses. The power was set to 0.80 and the minimum relevant difference in calcium deposition was set to 1 mM between study groups.

Normality of data was confirmed by QQ-plots (SAS Institute Inc., Cary, NC, USA). The differentiation data, comparing age groups within cell types and across cell types, were analyzed with a generalized linear mixed model using The GLIMMIX Procedure in SAS 9.4 (SAS Institute Inc., Cary, NC, USA) with Tukey–Kramer’s post hoc modifications for multiple comparisons. Statistical analysis of Fluidigm RT-qPCR results was performed on individual extracted Ln(RQ) values. Genes with missing datum points due to lack of detectable expression (as visualized in Additional file 1) were removed from the statistical analysis for the given cell type. Gene expression data were analyzed in two steps. Initially, one-way analysis of variance (ANOVA) using SAS 9.4 was applied individually to eighty-eight gene targets with measurable gene expression within each cell type to look for donor age effects. Next, targets showing significant difference on the ANOVA and more than five-fold difference between study groups on the heatmap (Additional file 1) were analyzed using The GLIMMIX Procedure with Tukey–Kramer’s post hoc modifications for multiple comparisons to compare across tissue types. To control for non-paired samples and potential inter-laboratory variables, horse number and laboratory were added to the statistical models as additional factors. Due to age-clustering within age groups, statistical linear regression analyses were not feasible and kinetics were determined based on graphical presentations. Data were considered statistically significant at *p*-values < 0.05.

## Supplementary Information

**Additional file 1.**

## Data Availability

Datasets generated and analyzed during this study are included in this published article and additional file. The accompanying source data and supplementary information are available from the corresponding author upon reasonable request.

## Abbreviations

ALP: Alkaline phosphatase
ANOVA: Analysis of variance
ARS: Alizarin Red S
AT: Adipose tissue
BM: Bone marrow
BSA: Bovine serum albumin
Ct: Cycle threshold
DF: Dermal fibroblast
DMEM: Dulbecco’s modified Eagle’s medium
dPBS: Dulbecco’s phosphate buffered saline
FBS: Fetal bovine serum
H&E: Hematoxylin and Eosin
IL-6: Interleukin-6
MSC: Mesenchymal stromal cell
polyHEMA: Poly 2-hydroxyethyl methacrylate
P/S: Penicillin/streptomycin
rhTGF-β1: Human recombinant transforming growth factor β1
RIN: RNA integrity number
RQ: Relative expression
RT-qPCR: Real-time quantitative PCR
