# Supplementary material for: Donor age effects on in vitro chondrogenic and osteogenic differentiation performance of equine bone marrow- and adipose tissue-derived mesenchymal stromal cells
Source: BMC Vet Res. 2022 Nov 3;18:388. doi: 10.1186/s12917-022-03475-2 (PMC9632053; doi:10.1186/s12917-022-03475-2)
Supplement: Supplementary file 1 — Additional file 1. [file 12917_2022_3475_MOESM1_ESM.docx]

**Additional file 1: Heatmap showing gene expression of eighty-eight biomarkers.**


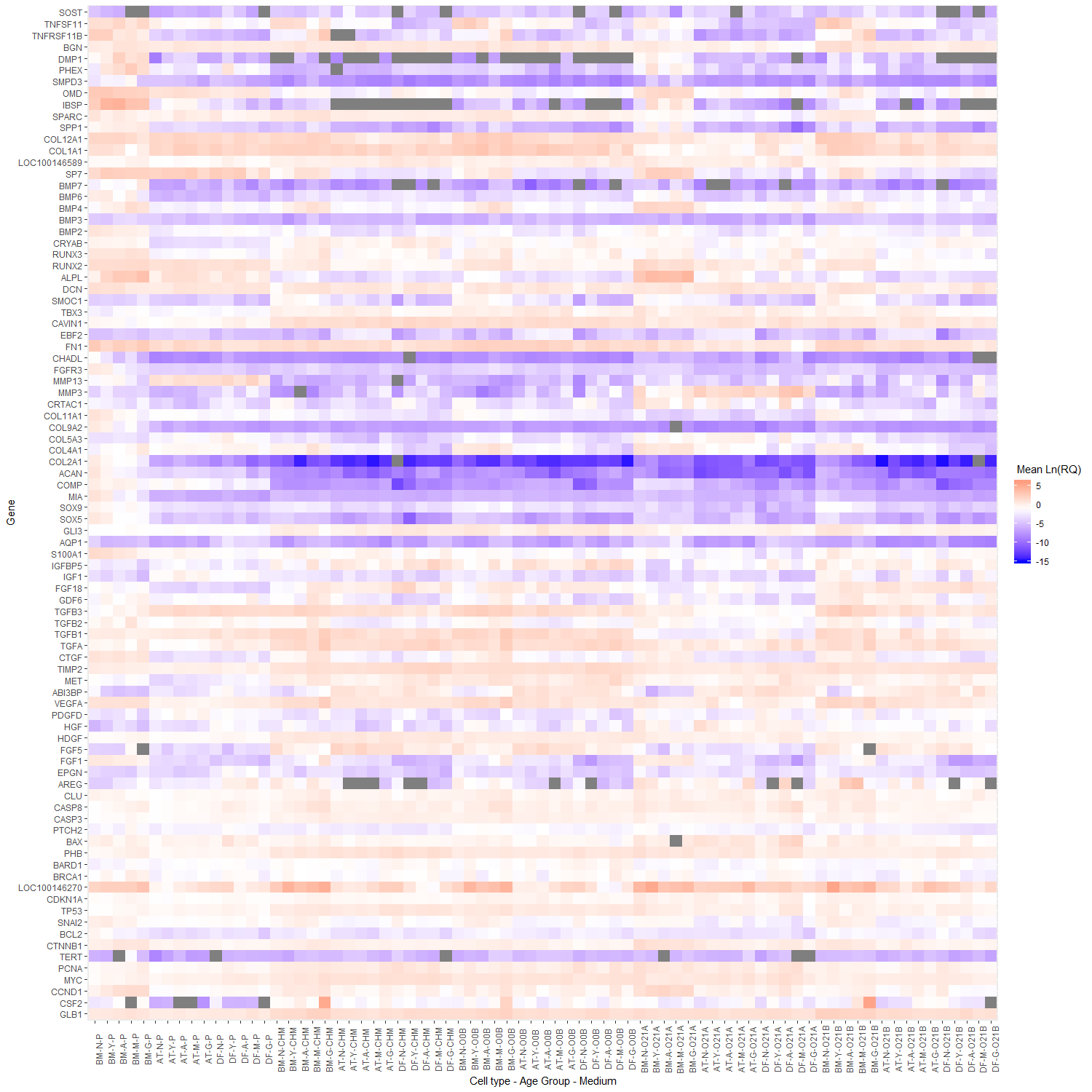


*A heatmap showing the Ln(RQ) levels of gene expression by color change between average levels in bone marrow (BM)- and adipose tissue (AT)-derived mesenchymal stromal cells and dermal fibroblasts (DF) from horses in five different age groups (n= 4 horses per age group per cell type per medium) grown under different conditions. Gray boxes indicate no detection of gene expression. ^†^N: Newborn, Y: Yearling, A: Adult, M: Middle-aged, G: Geriatric, P: Pellets cultured in chondrogenic induction medium for 21 days, CHM: grown in T75 flasks with expansion medium and harvested at ~80% confluence, O0B; grown in expansion medium in 6-wells and harvested at ~90% confluence, O21A: cultured in 6-wells in osteogenic induction medium for 21 days, O21B: cultured in 6-wells in expansion medium for 21 days.*
